# Malignant Arrhythmias Caused by Pericardial Lipomas: Benign, Yet Deadly

**DOI:** 10.1016/j.jaccas.2026.110316

**Published:** 2026-09-16

**Authors:** Matthew Henriques, Farah Tamizuddin, Victor Becerra-Gonzales, Brian Hsia, Kana Fujikura, Jill Jacobs, Robert Donnino, Dan Halpern, Aubrey Galloway, Thittamaranahalli Kariyappa Susheel Kumar, Lior Jankelson, Chirag Barbhaiya

**Affiliations:** NYU-Langone Medical Center, New York, New York, USA

**Keywords:** cardiac arrest, lipoma, ventricular tachycardia

## Abstract

**Background:**

Cardiac lipomas are usually benign but can cause significant hemodynamic and arrhythmic complications, such as ventricular tachycardia, depending on size, location, and myocardial involvement.

**Case 1 Summary:**

An 18-year-old man presented with cardiac arrest during exercise. Following a normal coronary angiogram, a defibrillator was implanted. After recurrent shocks, imaging demonstrated a large cardiac lipoma. Surgical excision resulted in cessation of his recurrent ventricular tachycardia.

**Case 2 Summary:**

A 25-year-old woman presented with ventricular tachycardia, and imaging revealed a large cardiac lipoma with coronary artery encasement. Surgical resection led to resolution of ventricular arrhythmias, and permanent defibrillator implantation was avoided.

**Discussion:**

These cases highlight how cardiac lipomas can lead to ventricular arrhythmias through mass effect and proximity to the coronary vasculature. Multimodal imaging was indispensable for the diagnosis and surgical planning for these cases.

**Take-Home Messages:**

Cardiac lipomas can cause life-threatening ventricular arrhythmias. A multimodal imaging approach combined with multidisciplinary team evaluation is essential for accurate diagnosis and management.

## Visual Summary

**Figure:**
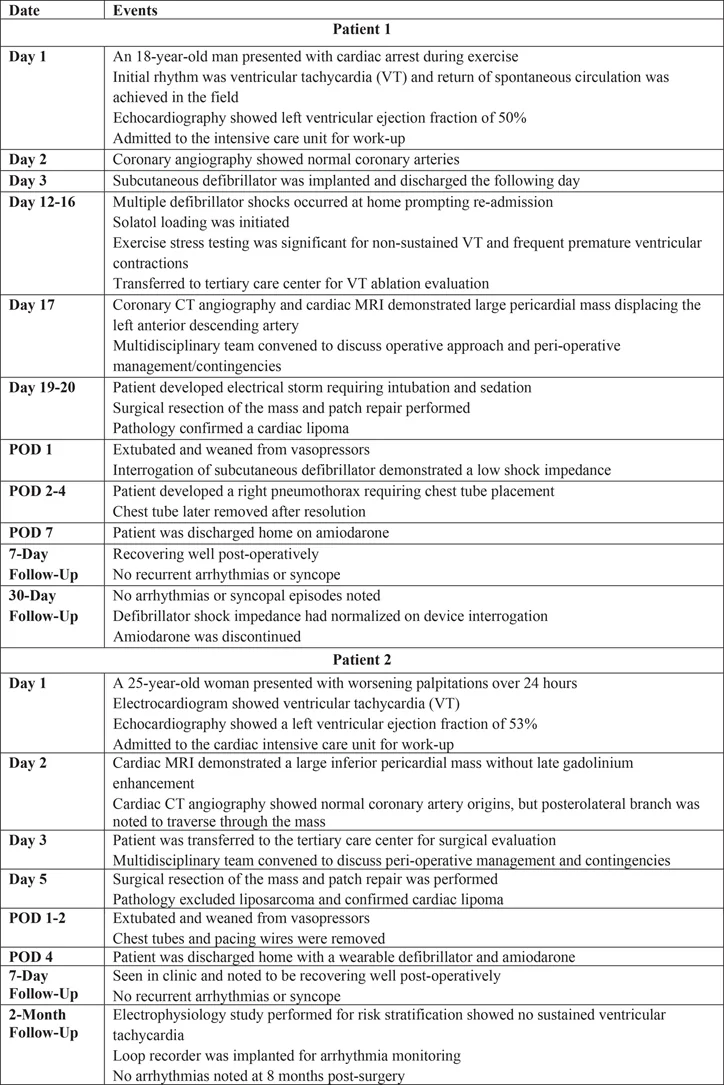

Cardiac lipomas are rare, comprising 8.4% of benign cardiac tumors.^1^ They may arise from the endocardium, myocardium, or subepicardium, and are commonly identified in the right atrium, left ventricle, cardiac valves, and pericardium.^2^, ^3^, ^4^ Ventricular arrhythmias in young patients without cardiomyopathies are often benign, commonly originating from the outflow tracts or fascicles.^5^ Ventricular arrhythmias from cardiac lipomas are well-described and may be related to ischemia from coronary artery compression, myocardial infiltration, mechanical irritation, or tethering of the ventricular wall.^1^^,^^2^^,^^6^^,^^7^ We present 2 cases of young patients with large pericardial lipomas associated with ventricular tachycardia, highlighting the diagnostic challenges and multidisciplinary management considerations.

## Case 1

### Patient presentation

An 18-year-old man with no prior medical history presented after cardiac arrest at the gym following a workout. Bystander cardiopulmonary resuscitation was initiated, and a defibrillator delivered 1 shock for ventricular tachycardia (VT) before first responders arrived. He required 3 additional shocks for VT before achieving sinus rhythm with return of spontaneous circulation and intact mental status.

On arrival to the outside emergency department (ED), he was tachycardic with otherwise stable vitals. He denied supplement use aside from creatine, denied recent illnesses, illicit drug use, or any family history of sudden cardiac death (SCD).

His initial electrocardiogram (ECG) showed sinus tachycardia (Figure 1A). Initial cardiac biomarkers were normal, electrolytes were normal, and a urine drug screen was negative. A chest radiograph showed clear lungs and mildly prominent heart size. Genetic testing for inherited arrhythmia syndromes was sent.

**Figure 1:**
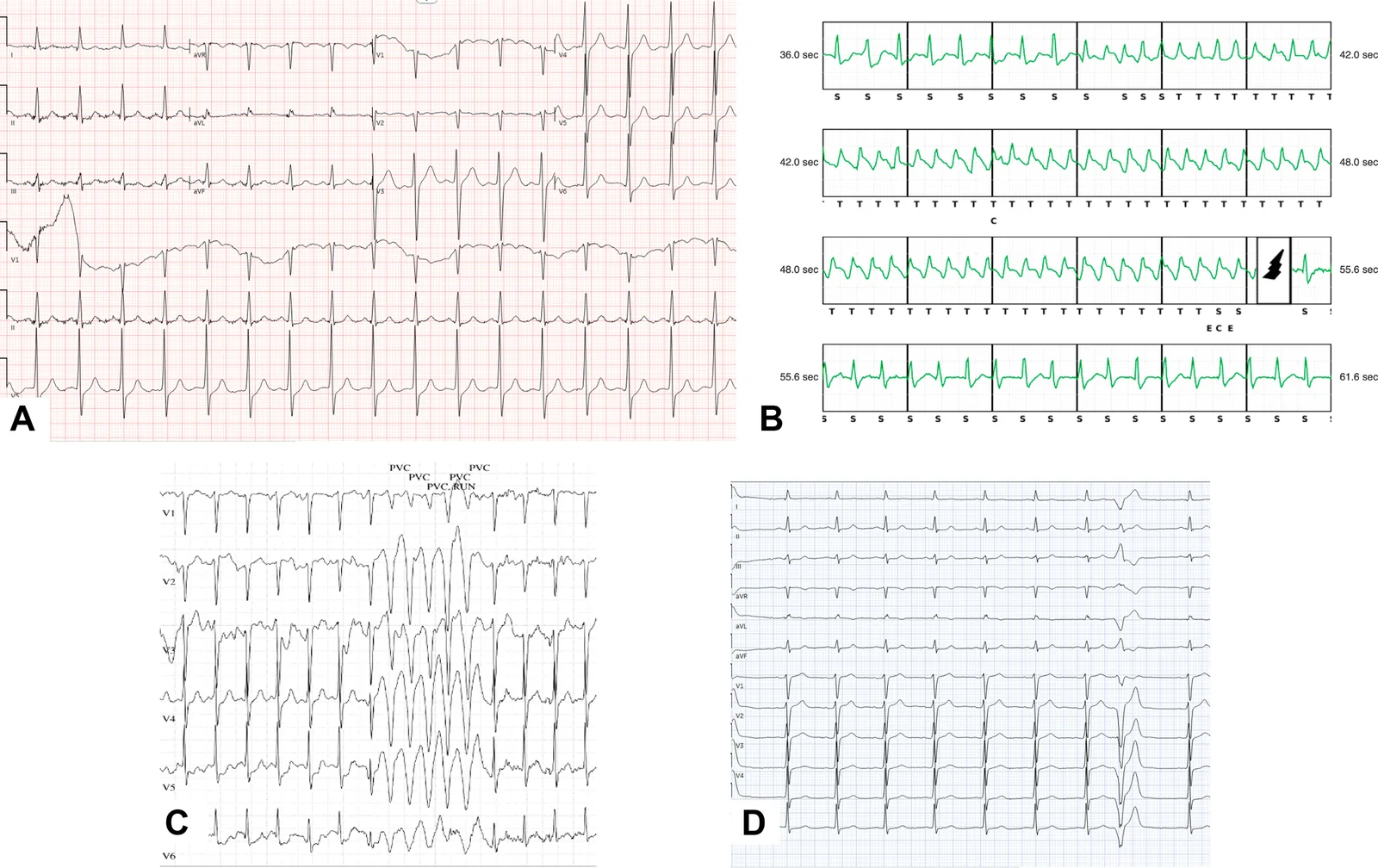
Patient 1 Electrocardiograms and Electrograms Patient 1 (A) Presenting electrocardiogram (ECG) demonstrating sinus rhythm. (B) Initial ventricular tachycardia (VT) episode on subcutaneous implantable cardioverter-defibrillator (S-ICD) with initiation, detection, shock, and conversion to sinus rhythm. (C) Nonsustained polymorphic VT during stress test. (D) ECG with premature ventricular contractions (PVCs) of apical anterior RV origin.

### Diagnostic work-up

He was admitted to the intensive care unit for workup. A transthoracic echocardiogram demonstrated a left ventricular (LV) ejection fraction of 50%, without structural or valvular abnormalities. A coronary angiogram demonstrated normal coronary arteries. Dedicated cardiac magnetic resonance imaging (MRI) was unavailable; therefore, it was scheduled as an outpatient, and a subcutaneous (Boston Scientific) defibrillator was implanted. Antiarrhythmic agents were discontinued on discharge.

Twelve days post-discharge, his defibrillator shocked him 3 times within 7 minutes during a phone argument. He re-presented to the outside ED where an interrogation demonstrated appropriate therapy for VT (Figure 1B). Sotalol 120 mg twice daily was initiated. He underwent ECG stress testing to evaluate sympathetically-mediated VT, during which he achieved 13 metabolic equivalents of task. Stress testing showed no ischemia but demonstrated 6 beats of nonsustained polymorphic VT despite sotalol loading (Figures 1C). Infrequent premature ventricular contractions were captured occasionally on ECG (Figure 1D). Given his recurrent VT, he was transferred to our institution for cardiac MRI and possible VT ablation.

Cardiac computed tomography angiography (CCTA) showed a 3.6 cm fat density mass along the anterior aspect of the mid–apical LV, abutting the mid–left anterior descending (LAD) artery with adjacent thinning of the myocardium (Figures 2A and 2B). Cardiac MRI confirmed a 4.2 × 2.2 cm lipoma (size variation from imaging plane differences) over the anterior LV (Figure 2C), with a left ventricular ejection fraction (LVEF) of 52%, a right ventricular ejection fraction (RVEF) of 43%, and no definitive abnormal late gadolinium enhancement (LGE) despite defibrillator artifact. The mass appeared to displace the LAD, possibly compressing a small septal branch, causing thinning of the LV myocardium to 2 mm in diastole. Positron emission tomography–computed tomography showed no evidence of hypermetabolic activity, consistent with a lipoma.

**Figure 2:**
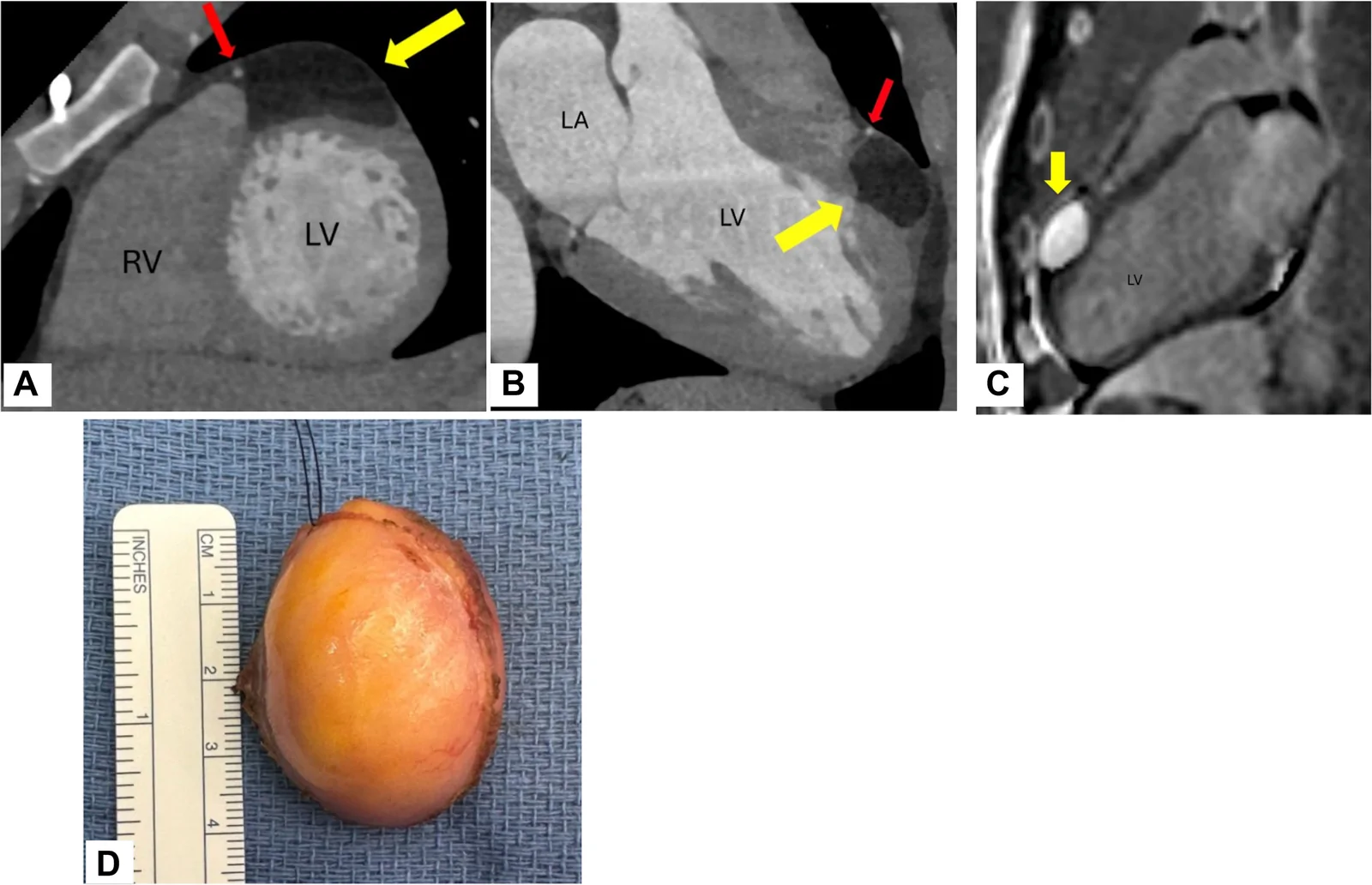
Patient 1 Imaging and Gross Specimen Patient 1 (A and B) Coronary computed tomography angiography (CTA) short-axis and modified 3-chamber reconstructions showing fat-density mass (yellow arrows) along the anterior wall of the left ventricle. The mass abuts yet does not encase or narrow the left anterior descending coronary artery (red arrows). (C) Two-chamber late-gadolinium enhanced image showing fat-intensity lipoma (yellow arrow) along the anterior wall of the left ventricle. There is no late gadolinium enhancement of the myocardium. (D) Excised pericardial lipoma. LA = left anterior; LV = left ventricular; RV = right ventricular.

A multidisciplinary plan was made to resect the mass and reconstruct the LAD with pericardial patching of the thinned LV. While awaiting surgery, he developed an electrical storm with multiple defibrillator shocks refractory to antiarrhythmic agent, and ultimately required intubation and sedation for VT suppression.

## Interventions

On hospital day 10, he underwent surgical intervention via midline sternotomy on cardiopulmonary bypass. The tumor abutted the LAD and ramus intermedius arteries, and was successfully excised with electrocautery dissection (Figure 2D). Pathology confirmed the diagnosis of lipoma. The residual myocardial compressive defect was repaired with a Dacron patch and a bovine pericardial patch. The defibrillator impedance was <30 Ω postoperatively.

Postoperatively, he developed a right pneumothorax, which resolved on postoperative day #4. Psychiatry was consulted for posttraumatic stress disorder symptoms in the setting of his extensive hospital course and multiple shocks. He continued amiodarone until follow-up.

## Outcome

The cardiogenetics panel demonstrated a likely pathogenic variant in the titin gene (c.17718+1G>T) associated with dilated cardiomyopathy, and variants of undetermined significance (VUS) in CACNB2, HCN4, and SNTA1. At his 6-week postoperative visit, his defibrillator impedance normalized to 45 Ω, amiodarone was discontinued, and metoprolol was continued. An outpatient cardiac monitor showed no episodes of VT and infrequent premature ventricular contractions. He completed cardiac rehabilitation 8 months postoperatively without further VT or syncopal episodes. He continues to have quarterly remote device interrogations, serial echocardiography, and regular ongoing cardiology follow-up.

## Case 2

### Patient presentation

A 25-year-old woman with no prior medical history presented to urgent care with 24 hours of palpitations. She was found to have a pulse of 154 beats/min with wide-complex tachycardia on ECG. Intravenous adenosine was ineffective; therefore, she was sent to the ED.

On ED presentation, her pulse was 162 beats/min with otherwise normal vital signs. An ECG showed VT with an apical-inferior right ventricular exit site terminating in sinus rhythm (Figure 3A). The sinus rhythm QRS interval showed a small J wave (Figures 3A and 3B). A subsequent ECG demonstrated initiation of another VT with a mid–apical inferior LV origin (Figure 3B). She endorsed transient chest fluttering over the preceding 2 months and nasal congestion from seasonal allergies for 1 week. She denied chest pain, shortness of breath, or syncope. She had no family history of SCD but reported a family history of early myocardial infarction. She denied smoking, alcohol use, supplement use, and drug use. Her only medication was an oral contraceptive.

**Figure 3:**
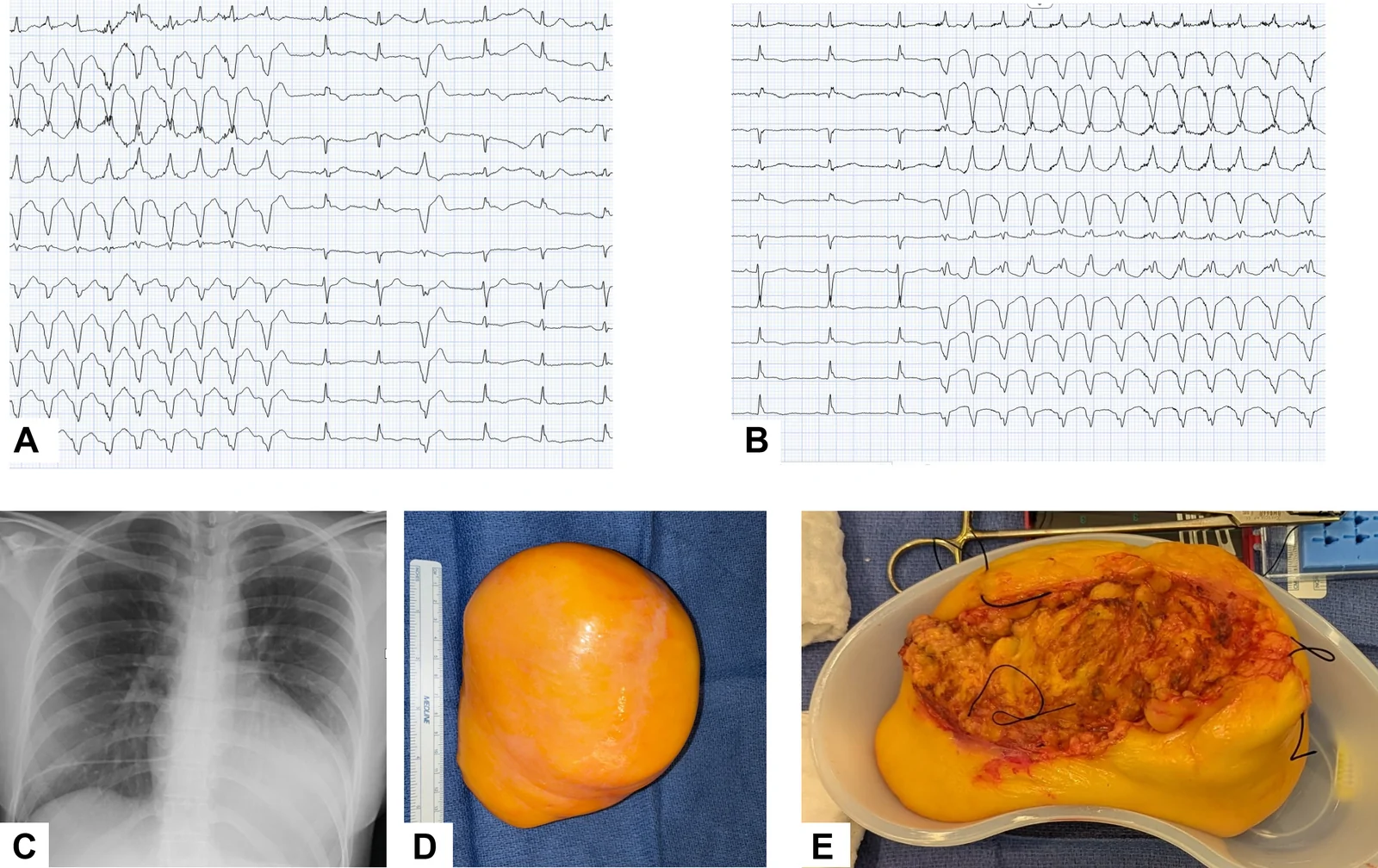
Patient 2 Electrocardiograms, Imaging, and Gross Specimens Patient 2 (A) Initial electrocardiogram (ECG) with ventricular tachycardia (VT) of apical inferior origin terminating to sinus rhythm. (B) ECG with resumption of nearby VT with apical inferior origin. (C) Chest radiograph with left pleural effusion and silhouetting of the left cardiac border. (D and E) Postsurgical excision of specimen showing smooth external surface and irregular surface of the lipoma adjacent to the left ventricular (LV) myocardium.

Initial labs were unremarkable. A chest radiograph demonstrated a small-moderate left pleural effusion, patchy opacities at the left base and retrocardiac region, and left cardiac border silhouetting (Figure 3C).

She was given intravenous metoprolol and a bolus of amiodarone before starting an amiodarone infusion. She was then admitted to the cardiac intensive care unit for monitoring.

### Diagnostic work-up

A transthoracic echocardiogram demonstrated a LVEF of 53%, normal left and right heart morphology and function, normal LV end-diastolic diameter of 4.9 cm, and no significant valvular abnormalities. Notably, the apex was not well-visualized.

In preparation for possible VT ablation, a cardiac MRI was performed, showing a pericardial lipomatous mass measuring 12 × 10 × 12 cm (Figures 4A to D). The mass was located along the inferior and inferolateral walls of the LV and the inferior aspect of the RV. Strands of myocardial tissue appeared to extend into the mass, emanating from the LV subepicardium inferiorly. The LV inferior wall showed thinning and mild hypokinesis with an LVEF of 67% and a normal RVEF. No LGE of the myocardium or inflammation/edema on T2 imaging were noted. The findings were consistent with a lipoma.

**Figure 4:**
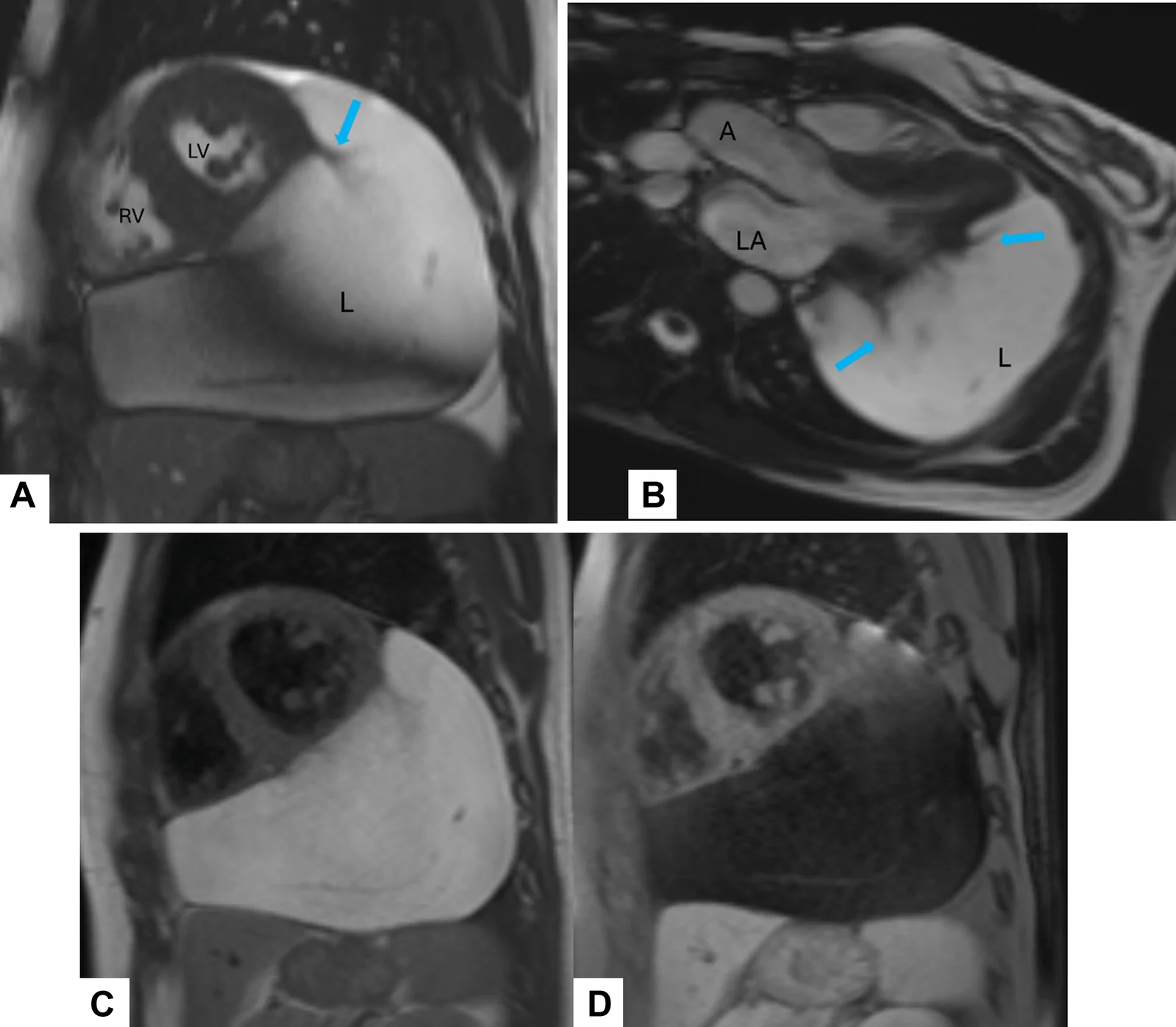
Patient 2 Magnetic Resonance Imaging Patient 2 (A and B) Short-axis and 3-chamber steady-state free precession magnetic resonance imaging (MRI) images showing a large lipomatous mass along the inferior cardiac border and inferolateral left ventricle. Myocardial thinning with strands of presumed myocardial tissue (blue arrows) is seen emanating from the inferior/inferolateral walls. Myocardial thinning is seen in the inferolateral wall. (C and D) T1-weighted imaging without fat saturation and with fat saturation, respectively. A = aorta; L = lipoma; other abbreviations as in Figure 2.

Salvos of nonsustained VT continued, and she was started on a lidocaine infusion before transfer to our institution. On CCTA, the mass appeared attached to the LV epicardium on the basal, inferolateral, and inferior segments with thinning of the walls and myocardial strands extending into the mass (Figures 5A and 5B). The mass abutted the posterior wall of the RV from base to apex with a clear border between the mass, the ventricle, and the pericardium. Coronary arteries demonstrated normal origins. The posterolateral branch of the left circumflex coronary artery traveled through the mass, directing back to the myocardial and epicardial surface of the LV at the apical inferolateral segment (Figure 5B). The coronary branch traversing through the mass raised concern for ischemia via mass effect on the artery or a steal effect from the mass. The presence of myocardial projections extending to the mass led to consideration of liposarcoma; however, this was felt less likely, and further imaging with positron emission tomography–computed tomography was deemed unlikely to definitively exclude malignancy. A multidisciplinary plan was made for mass resection and patch reconstruction of the ventricular myocardium. Backup mechanical support was planned as a bridge-to-transplant in the event of perioperative complications.

**Figure 5:**
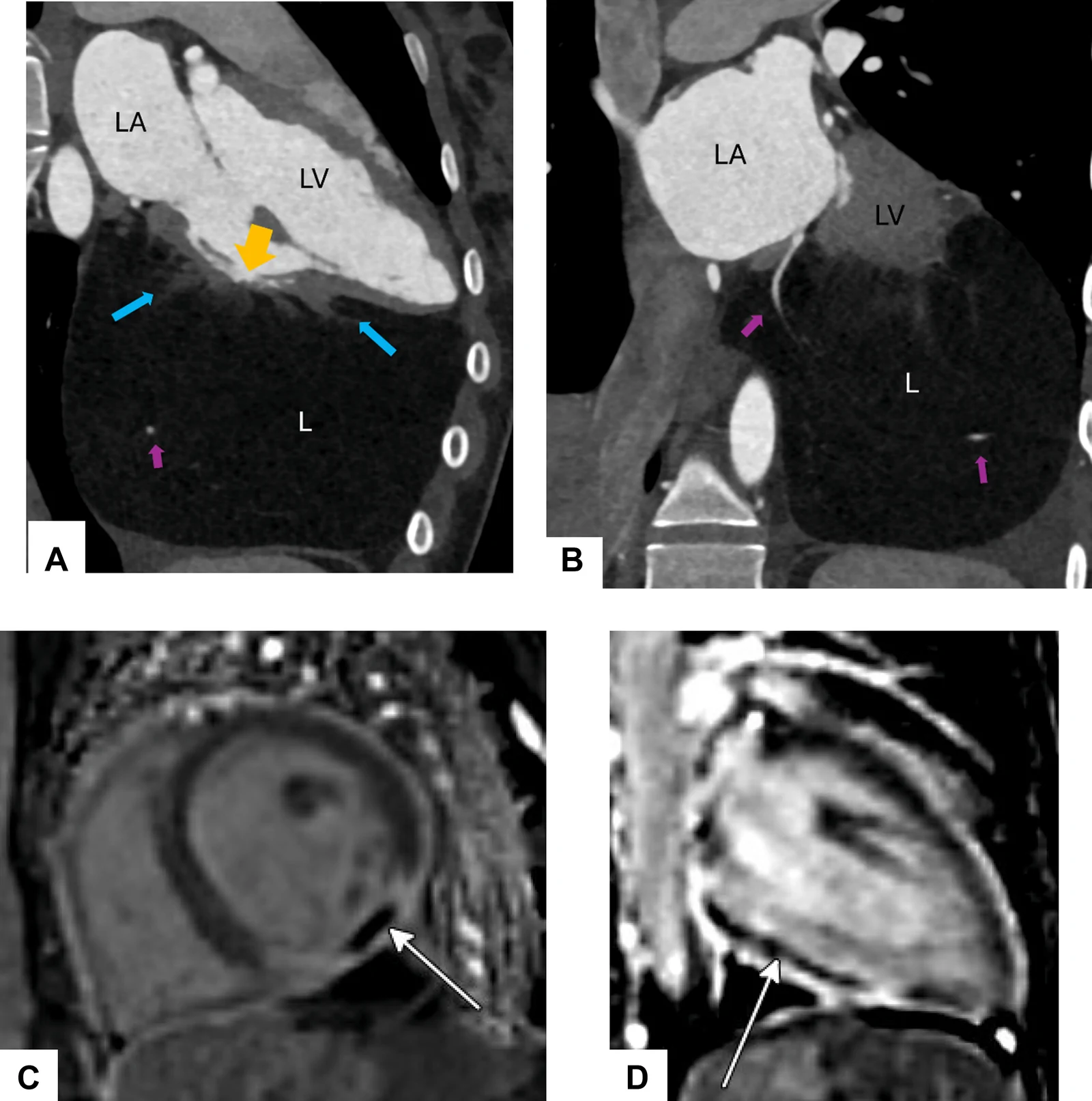
Patient 2 Cardiac Computed Tomography Angiography Imaging Patient 2 (A and B) Coronary computed tomography angiography (CTA) multiplanar reformatted images showing thinning of the left ventricular myocardium (orange arrow) and strands of presumed myocardial tissue (blue arrows) along the inferior/inferolateral walls extending into the large lipoma. The left posterolateral coronary vessel extends into the lipoma (purple arrow). (C and D) Postoperative magnetic resonance imaging (MRI) images depicting postsurgical transmural late gadolinium enhancement (LGE) in the basal-mid inferior left ventricular (LV). A = aorta; L = lipoma; other abbreviations as in Figure 2.

## Interventions

Surgical intervention occurred 5 days after presentation via median sternotomy with cardiopulmonary bypass. The tumor was dissected along the free margin of the RV toward the inferior LV using electrocautery. The mass was separated medially from the right atrium/inferior vena cava. The central segment of the mass originated from the inferior LV, near the posterior descending artery and atrioventricular groove. The heart was reflected rightward and superiorly to expose the lateral insertion, which was then dissected medially. The posterolateral branch was ligated, and the mass was dissected from the coronary sinus and distal circumflex artery. The inferior segment of the mass (5 × 4 cm, Figures 3D and 3E) was then dissected, requiring intramyocardial dissection. The mass was removed en bloc and sent for pathology analysis. Sutures and a bovine pericardial patch were applied, and the coronary arteries were inspected for bleeding before weaning her from bypass. Fluorescence in situ hybridization studies were negative for MDM2, excluding well-differentiated liposarcoma, and confirming the diagnosis of cardiac lipoma.

On postoperative day #2, she was ambulatory, her chest tubes were removed, and her J-wave was no longer present on ECG. Electrophysiology (EP) recommended continuation of amiodarone 200 mg, a wearable cardiac defibrillator, and an outpatient EP study to assess for inducibility of VT and to risk stratify for SCD. Cardiac genetic testing showed VUS in AKAP9, FKRP, and SCN10A.

## Outcome

Colchicine was later initiated for postpericardiotomy syndrome. At her EP follow-up visit, amiodarone was discontinued. A postsurgical cardiac MRI (Figures 5C and 5D) showed normal LVEF and RVEF, and akinesis of the midinferior/inferolateral segments correlating with tumor resection sites. A low-intensity area representing the patch was also surrounded by transmural LGE representing postsurgical scar. Two months later, an EP study demonstrated no inducible, sustained VT, though ventricular fibrillation did occur with tight-coupled triple extrastimuli. Her wearable defibrillator was discontinued, and an implantable loop recorder was inserted for long-term monitoring. She remains free of significant arrhythmias at 1 year.

## Discussion

These cases highlight dramatic presentations in which cardiac lipomas caused ventricular arrhythmias. Although lipomas are histologically benign, their clinical impact may vary based on proximity to surrounding cardiac structures. There are currently no specific guidelines from the American College of Cardiology or American Heart Association regarding the management of ventricular arrhythmias secondary to pericardial masses.

Although the mechanism of VT in the setting of pericardial masses is not fully elucidated, the lack of LGE on presurgical MRI and pleomorphic VT morphologies in these cases suggest that scar-mediated re-entry is less likely. In both cases, marked thinning of the LV myocardium adjacent to the lipomas was noted, but the absence of LGE in these areas favors thinning due to mechanical impingement given no detectable scar. Myocardial tissue extending into the lipoma has been described previously.^8^ Interestingly, the ECG of each patient's VT suggested origins localizing to the anatomic tumor locations.

The genetic panel in case 1 demonstrated a “likely pathogenic” intron splice site variant not reported in databases. The clinical significance of this variant remains uncertain; however, in the absence of dilated cardiomyopathy or LGE, primary ventricular arrhythmia due to subclinical disease from this variant is very unlikely. In case 2, the genetic panel included a VUS in SCN10A that leads to loss-of-function. There are reports of SCN10A truncating variants being associated with ventricular arrhythmias^9^ and sudden death;^10^ however, studies of channel blockade suggest attenuation of ventricular arrhythmias in canine models.^9^ Although these patients had genetic variants that could be associated with arrhythmias, the resolution of VT in both patients (as well as the J-wave in patient 2) after resection strongly implicates the lipomas as causal to arrhythmogenesis.

## Conclusions

In young patients presenting with ventricular arrhythmias without structural abnormalities on echocardiography or angiography, imaging with cardiac MRI and CCTA can reveal occult findings, including cardiac tumors. Even histologically benign cardiac tumors can cause ventricular arrhythmias and cardiac arrest; therefore, it is important to characterize the etiology of VT and treat the underlying cause. As in our first case, secondary prevention defibrillator implantation may be performed before the identification of an underlying cause, particularly when advanced imaging availability is limited. However, as in our second case, tumor resection may obviate the need for defibrillator implantation. Given the rarity of these cases and the lack of specific guidelines, a multidisciplinary approach and close clinical monitoring are essential to optimizing patient outcomes.

## Funding Support and Author Disclosures

The authors have reported that they have no relationships relevant to the contents of this paper to disclose.

**Take-Home Messages**

• Advanced imaging can help elucidate potential mechanisms for ventricular tachycardia, particularly in young patients.
• Cardiac lipoma size does not always correlate with the clinical severity of associated ventricular arrhythmias.
• The management of pericardial masses that cause clinical arrhythmias benefits from a multidisciplinary approach to optimize surgical outcomes.

**Visual Summary:** Timelines of Sequential Events From Patient 1's and Patient 2's Initial Clinical Presentations to Postdischarge Follow-Up Appointments

Date	Events
Patient 1	
D 1	An 18-year-old man presented with cardiac arrest during exercise. Initial rhythm was VT, and return of spontaneous circulation was achieved in the field. Echocardiography showed a left ventricular ejection fraction of 50%. Admitted to the intensive care unit for workup.
D 2	Coronary angiography showed normal coronary arteries.
D 3	A subcutaneous defibrillator was implanted and he was discharged the following day.
D 12-16	Multiple defibrillator shocks occurred at home, prompting readmission. Solatol loading was initiated. Exercise stress testing was significant for nonsustained VT and frequent premature ventricular contractions. He was transferred to a tertiary care center for VT ablation evaluation.
D 17	Coronary CT angiography and cardiac MRI demonstrated a large pericardial mass displacing the left anterior descending artery. A multidisciplinary team convened to discuss the operative approach and perioperative management/contingencies.
D 19-20	Patient developed an electrical storm requiring intubation and sedation. Surgical resection of the mass and patch repair were performed. Pathology confirmed a cardiac lipoma.
POD 1	Extubated and weaned from vasopressors. Interrogation of the subcutaneous defibrillator demonstrated a low shock impedance.
POD 2-4	Patient developed a right pneumothorax requiring chest tube placement. The chest tube was later removed after resolution.
POD 7	The patient was discharged home on amiodarone.
7-D follow-up	Recovering well postoperatively. No recurrent arrhythmias or syncope.
30-D follow-up	No arrhythmias or syncopal episodes noted. Defibrillator shock impedance had normalized on device interrogation. Amiodarone was discontinued.
Patient 2	
D 1	A 25-year-old woman presented with worsening palpitations over 24 h. An electrocardiogram showed VT. Echocardiography showed a left ventricular ejection fraction of 53%. Admitted to the cardiac intensive care unit for workup.
D 2	Cardiac MRI demonstrated a large inferior pericardial mass without late gadolinium enhancement. Cardiac CT angiography showed normal coronary artery origins, but the posterolateral branch was noted to traverse through the mass.
D 3	Patient was transferred to the tertiary care center for surgical evaluation. A multidisciplinary team convened to discuss perioperative management and contingencies.
D 5	Surgical resection of the mass and patch repair were performed. Pathology excluded liposarcoma and confirmed cardiac lipoma.
POD 1-2	Extubated and weaned from vasopressors. Chest tubes and pacing wires were removed.
POD 4	Patient was discharged home with a wearable defibrillator and amiodarone.
7-D follow-up	Seen in clinic and noted to be recovering well postoperatively. No recurrent arrhythmias or syncope
2-Mo follow-up	Electrophysiology study performed for risk stratification showed no sustained ventricular tachycardia. Loop recorder was implanted for arrhythmia monitoring. No arrhythmias noted at 8 mo post-surgery.

CT = computed tomography; MRI = magnetic resonance imaging; POD = postoperative day; VT = ventricular tachycardia.
